# Nanojunction Effects on Water Flow in Carbon Nanotubes

**DOI:** 10.1038/s41598-018-26072-6

**Published:** 2018-05-17

**Authors:** Fatemeh Ebrahimi, Farzaneh Ramazani, Muhammad Sahimi

**Affiliations:** 1 Physics Department, University of Birjand, Birjand, 97175-615 Iran; 2 Mork Family Department of Chemical Engineering and Materials Science, University of Southern California, Los Angeles, California 90089-1211 USA

## Abstract

We report on the results of extensive molecular dynamics simulation of water imbibition in carbon nanotubes (CNTs), connected together by converging or diverging nanojunctions in various configurations. The goal of the study is to understand the effect of the nanojunctions on the interface motion, as well as the differences between what we study and water imbibition in *microchannels*. While the dynamics of water uptake in the entrance CNT is the same as that of imbibition in straight CNTs, with the main source of energy dissipation being the friction at the entrance, water uptake in the exit CNT is more complex due to significant energy loss in the nanojunctions. We derive an approximate but accurate expression for the pressure drop in the nanojunction. A remarkable difference between dynamic wetting of nano- and microjunctions is that, whereas water absorption time in the latter depends only on the ratios of the radii and of the lengths of the channels, the same is not true about the former, which is shown to be strongly dependent upon the size of each segment of the nanojunction. Interface pinning-depinning also occurs at the convex edges.

## Introduction

Flow of water in nanostructured materials^1^, and in particular in carbon nanotubes^2–14^ (CNTs) and their silicon-carbide^15–17^ counterparts, has been the subject of numerous theoretical, computational, and experimental studies. Both pressure-driven flow and spontaneous imbibition of water into such nanotubes have been studied. Many fascinating phenomena have been discovered, including water flow rates that are much larger than what the classical continuum hydrodynamics predicts, and water not freezing in small nanotubes at temperatures much below its bulk freezing temperature^14,15^. In addition, it was demonstrated that other factors, such as functionalization of the nanotubes, strongly influence water flow in CNTs^18–20^. There have also been studies of flow in various types of small capillaries whose atomistic structures are not, however, identical with that of CNTs. Das *et al*.^21^, for example, studied inviscid flow - one in which viscosity is unimportant and flow occurs due to the balance between the capillary and inertial effects - that invariably precedes the classical Washburn regime during capillary filling. Oyarzua *et al*.^22^ reported on a study of the effect of nano-confinement, the initial conditions of liquid uptake and air pressurization on the dynamics of capillary filling, identifying three main flow regimes in which the capillary force is balanced by (i) the inertial drag in the initial flow regime; (ii) both inertia and viscous friction in the transitional regime, and (iii) viscous forces alone. Fries and Dreyer^23^ considered the transition from inertial to viscous flow in capillary rise, focusing in particular on the early stages of the flow, while Hultmark *et al*.^24^ studied theoretically and by experiments the influence of a gas phase on liquid imbibition in long capillary tubes in which viscous resistance from the gas phase ahead of the moving front is significant. Kornev and Neimark^25^ studied spontaneous penetration of liquids into capillaries, and derived a generalized equation of the fluid front motion by averaging the Euler’s equation (inviscid flow) both inside and outside a capillary.

Implicit in the aforementioned papers^21,23–25^ are two main assumptions or approximations: (i) the no-slip boundary condition for fluid flow in the tubes, and (ii) neglecting the effect of liquid inertia on the dynamics of imbibition. The latter assumption is valid only in the long-time limit, when viscous friction becomes very large. More precisely, the early stage of capillary filling in microtubes is dominated by the inertia effect, resulting^26^ in a $L(t)\sim {R}^{-\mathrm{1/2}}t$ behavior for the length *L*(*t*) of the fluid column in the tube, where *R* is the tube’s radius, if we ignore the viscous resistance by the gas phase^24^. Therefore, in the case of short CNTs that we study in this paper, we need to modify the previous descriptions^21–25^ in order to take into account the giant slip length of water on the CNTs’ wall. The inertia effect can still be neglected, since there is a dissipation mechanism that is independent of the length of water column *L*(*t*).

However, with very few exception, all the studies so far have focused on pristine nanotubes (and micropores) with perfectly straight-channel geometry. Gravelle and co-workers^27,28^ and Tang *et al*.^29^ studied water flow through “hourglass” nanopores, i.e., two channels connected by a much smaller nanopore. Hanasaki and co-workers^13,14^ studied flow of water, as well as gases, through CNTs, but their focus was high-speed flow from the perspective of an intersection between a machine material and a device, which is completely different from what we study in the present paper. Study of water flow in complex nanofluidic structures that consist of interconnected nanotubes is, however, of both fundamental and practical interest. Theoretically, understanding how confinement and its geometry at nanoscale affect fluid flow and transport phenomena is not completely understood, and is a subject of many current studies. On the practical side, micro- and nanofluidic systems^5,30^, as well as nanoreactors^31–33^, are promising tools for improving not only the analysis of properties of biological, polymeric, and other types of materials, but also their synthesis, as they reduce the volume of fluid samples needed, and provide a well-controlled fluid environment for integrating various chemical processes.

Some recent studies demonstrated^34–39^ that simple changes in the cross-sectional area of microchannels may be used to regulate and optimize the velocity of capillary flow. Moreover, experimental techniques have been developed for fabricating axisymmetric circular nanotubes with varying cross sections, including buckling^40^ and plumbing^41^ of CNTs. The possibility of producing buckling of CNTs is due to their large aspect ratios and hollow geometry, which make them susceptible to structural instabilities under certain loading conditions. A more promising and controlled way of making CNT junctions, the plumbing CNTs, was proposed by Jin *et al*.^41^ who used electro-migration effects to join any two CNTs, regardless of their diameters.

It is, therefore, natural to study water imbibition and flow in CNTs with more complex geometries, including junctions that connect CNTs of various sizes, as one may use such configurations for optimizing the performance of nanofluidic and nanoreactor systems. In addition, the predominance of surface effects at the smallest length scales may lead to new phenomena and properties. In this paper we report on the results of extensive molecular dynamics (MD) simulations of water imbibition in CNTs with nanojunctions. In addition to its aforementioned significance, what we study is also relevant to many other phenomena, including water absorption into very tight pores in biological materials and very small living spieces. The emphasis is on the effect of the nanojunctions on flow of water in the CNTs, and the differences between what we study and flow of water in microchannels.

## Results

### The contact angle

As described in the Methods section, as well as in the Supplementary Information (SI), we computed the contact angle *θ* of a nanodroplet of water with the wall of the CNTs. We obtained, *θ* ≈ 55° ± 8° and 54° ± 7° in, respectively, the (20, 20) and (30, 30) CNTs. To see whether the structure of the nanojunctions makes any difference in the CA, we also computed *θ* for a configuration in which two (20, 20) CNTs are connected by a converging junction (see Fig. S4 in the SI). The CA turned out to be, *θ* ≈ 59° ± 8°. Thus, the CAs for the various configurations are consistent with each other, and are also in agreement with what has been reported in the literature, namely, *θ* ≈ 57°.

### Flow, dissipation and dynamics

**Figure 1:**
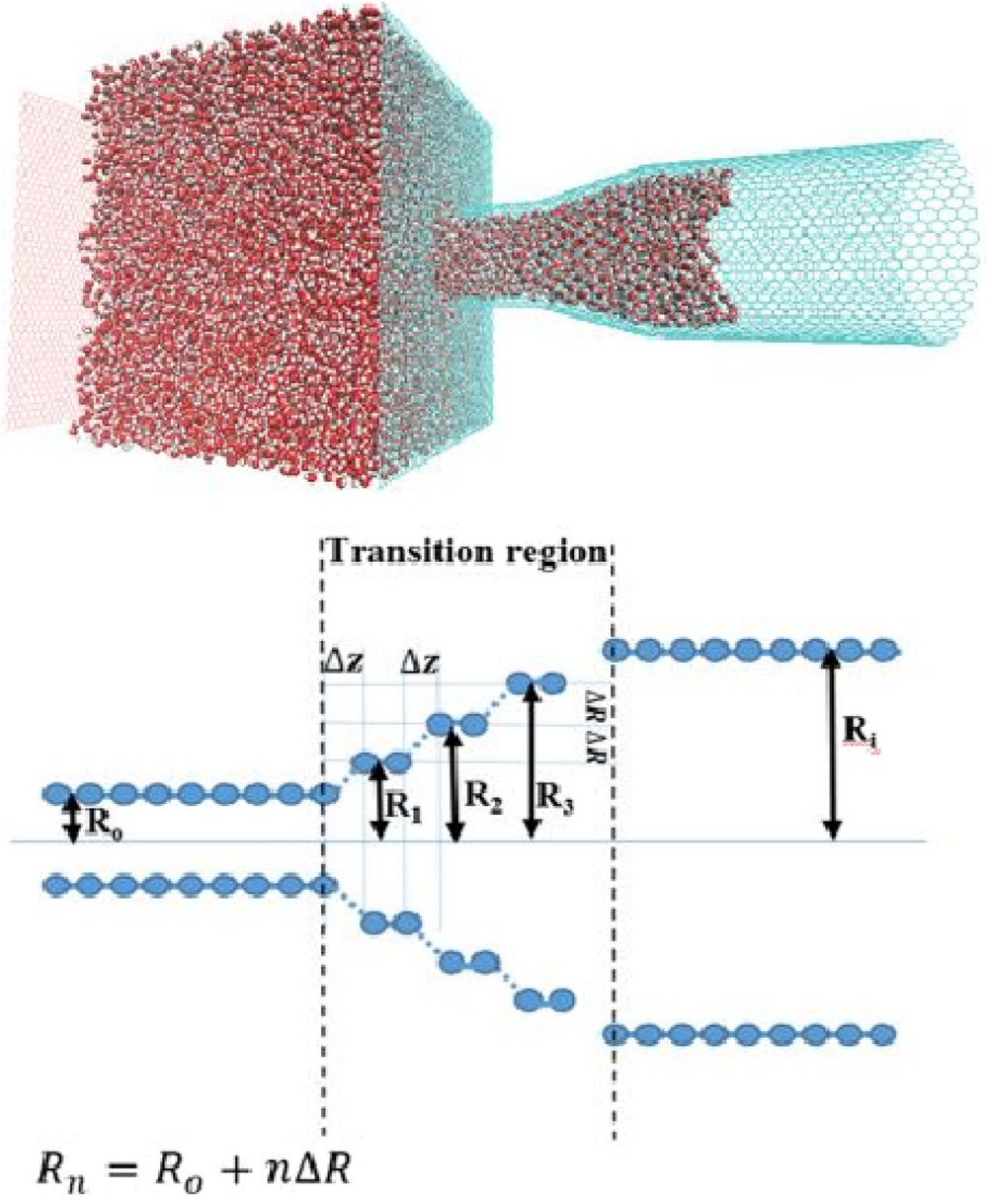
Schematic representation of the simulation system with a nanojunction and its geometry. Similar structures were used with two nanojunctions.

Figure 1 presents the schematic representation of the simulation system with a nanojunction and its geometry. Similar structures were used with two nanojunctions. The details of the MD simulations are described below. Before discussing the results, we should point out that application of the classical hydrodynamics to a similar problem in straight microchannels leads to the conclusion that the dynamical wetting is *faster* in wider tubes. The explanation is straightforward. In capillary action the Laplace pressure *P*_*c*_ that supplies the input power for spontaneous imbibition is proportional to the inverse of the tube’s radius *R*_*i*_ at the interface,

1 $${P}_{c}=2\frac{\gamma \,\cos \,\theta }{{R}_{i}}\equiv \frac{\beta }{{R}_{i}},$$

where *γ* is the surface tension of water and *θ* is the contact angle. On the other hand, the pressure drop resulting from viscous flow inside the same tube has a stronger dependence on the tube’s radius, namely,

2 $${\rm{\Delta }}P=\frac{8\eta L(t)Q}{\pi {R}_{o}^{4}},$$

where *R*_*o*_ is the radius of the tube (for a straight tube *R*_*i*_ = *R*_*o*_), *η* is the viscosity of water, *Q* is the volume flow rate, and *L*(*t*) is the distance between the reservoir’s fluid at the entrance to the tube and the interface. As such, a wider tube has a faster wetting, which also explains why the interface advancement accelerates for a short time after it enters the narrower segment of a converging microchannel, and then decelerates after travelling a long distance in the narrower (exit) nanotube. The same argument, when applied to a diverging junction, explains why the filling time is much larger than that of straight CNTs of the same length. In the case of microchannels, Reyssat *et al*.^42^ demonstrated that imbibition in a diverging geometry slows down very significantly, and that the details of the geometry affect the dynamics drastically, particularly at long times.

The situation is, however, quite different in nanochannels. In this regime MD simulations indicate that the rate of water advancement in short CNTs is almost independent of the size of the opening^26^. This feature is explained by noting that because of the very large slip length of water inside CNTs, friction at the walls of short CNTs should be completely negligible. As such, the main source of energy dissipation is the entrance friction corresponding to a pressure drop given by^43^

3 $${\rm{\Delta }}P=\frac{C\eta Q}{{R}_{o}^{3}},$$

where *C* is the loss coefficient. *Q* is the product of cross-sectional area and the fluid velocity, and at steady state the pressures given by Eqs (1) and (3) are equal, implying that $Q\propto {R}_{o}^{2}$. Therefore, the fluid velocity *dL*/*dt* and the position *L*(*t*) of the interface in all CNTs do not depend on their radius.

**Figure 2:**
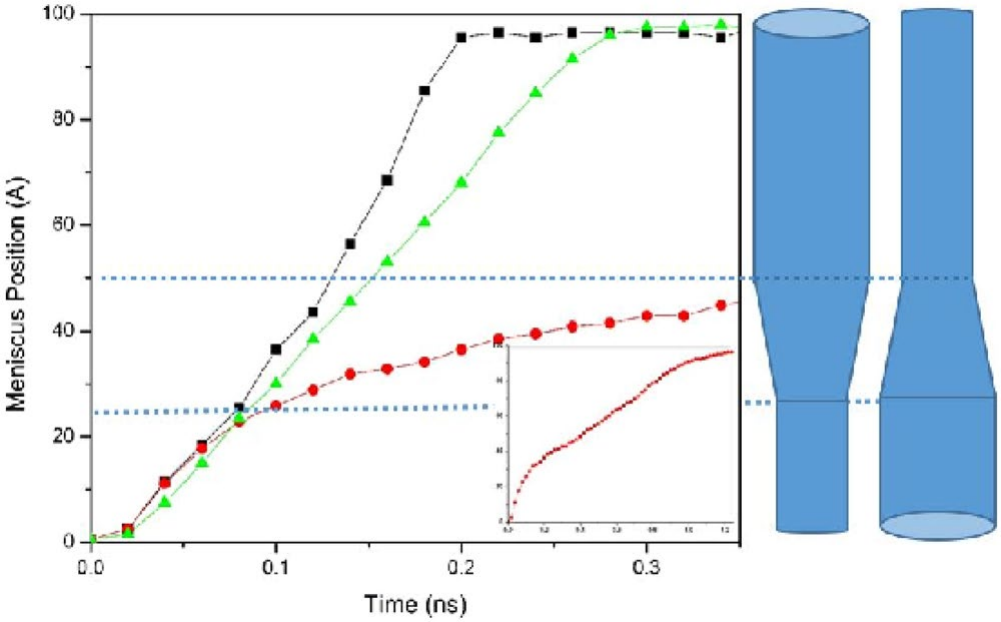
Dynamic evolution of the meniscus position in the converging (black squares) and diverging (red circles) nanojunctions that connect straight (20, 20) and (30, 30) CNTs. For comparison, we also show the corresponding position for a straight (30, 30) CNT of the same total length (green triangles). Inset shows the profile in the diverging geometry at short times.

We show in Fig. 2 the dynamic evolution of the interface position, or the meniscus level *L*(*t*), for the geometries shown in the figure. For comparison, we also show the corresponding quantity for a straight (30, 30) CNT of the same total length. Figure 2 indicates that the dynamics in the CNTs with a diverging junction is much slower than that in the straight CNT, whereas it is only a little faster in the CNTs with a converging junction. To explain the results for the nanotubes with a converging nanojunction, we note that as the fluid enters such a junction, *R*_*i*_ becomes smaller than *R*_*o*_, as a result of which the Laplace pressure is enhanced by a factor *R*_*o*_/*R*_*i*_. Moreover, another mechanism of energy dissipation in the converging nanojunction, namely, contraction of the streamlines, also contributes to the fluid flow. The two factors together explain why there is no acceleration of the interface when the fluid invades the nanojunction. Note, however, that, in general there is no reason for the two factors to cancel each out, as the hydraulic resistance as approximated here depends on the geometrical details of the nanojunction, whereas the Laplace pressure changes only with the size of opening at the place of three-phase contact line.

**Figure 3:**
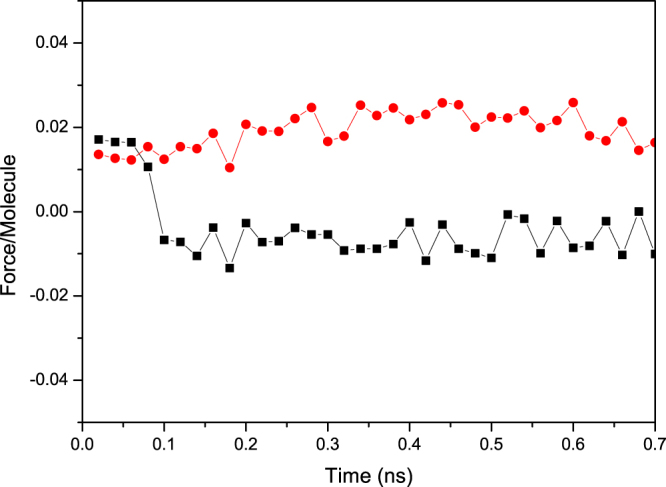
Average net force along the axial direction of the nanotubes exerted by the wall carbon atoms on each water molecules inside the nanotube in the converging *c*30–20 (black squares) and diverging *d*20–30 (red circles) geometries.

Figure 3 presents the average net axial force exerted by the CNT’s wall on each of the water molecules inside the nanotube. The important aspect of Fig. 3 is that, the net axial force in the CNTs with converging junction is negative; that is, it retards the flow. Note also that the number of carbon atoms per unit length in the converging junction decreases, and so also does the interaction energy between them and the water molecules. The negative force cannot, however, overcome the attraction between the oxygen atoms of water and the carbon atoms at or near the entrance of the nanotube. The net result is the advancement of the contact line through the nanojunction.

**Figure 4:**
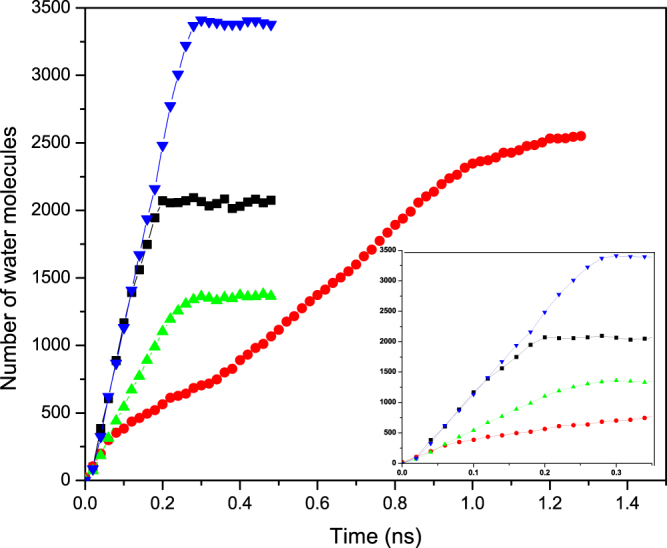
Time-dependence of the number of water molecules *N*(*t*) in the converging (black squares) and diverging (red circles) geometries with the 20–30 nanojunctions. Also shown are the corresponding quantities for a straight (20, 20) CNT (green up triangles) and (30, 30) CNT (blue down triangles) of the same total length. Inset shows the same profiles at short times.

For a more quantitative understanding of the phenomenon, we computed the time-dependence of the number of water molecules *N*(*t*) in the CNTs. The results are presented in Fig. 4. As one may anticipate, for both types of the CNTs the rate of uptake in the CNT connected to the water reservoir is almost equal to that of total uptake of a straight pristine CNT of the same radius. After passing the entrance nanotube, there is a transition regime in which the interface advances through the short nanotubes in the region that narrows or widens (depending on the type of the junction), followed by the final flow regime in which the front moves through the exit nanotube.

Let us first consider the final flow regime when the interface is moving through the exit CNT far from the nanojunction. As Fig. 4 indicates, the rate of water uptake changes almost abruptly after the interface leaves the transition region. If, similar to pristine CNTs, the entrance friction were the only source of energy dissipation, then, it would be straightforward to show that as the interface enters a channel of radius *R*_*i*_, the rate of water uptake is *Q*_*i*_ = (*R*_*o*_/*R*_*i*_)*Q*_*o*_, where *Q*_*o*_ is the uptake when fluid passes through the entrance straight CNT of radius *R*_*o*_. To see this, recall that at steady state the Laplace pressure *P*_*c*_ and the pressure drop Δ*P* given by Eq. (3) are equal. The equality, together with *R*_*i*_ = *R*_*o*_ for a straight CNT yields, ${Q}_{o}=\beta {R}_{o}^{2}/(C\eta )$. When water invades the second part of a nonstraight CNT of radius *R*_*i*_, we have, ${Q}_{i}=\beta {R}_{o}^{3}/(C\eta {R}_{i})=({R}_{o}/{R}_{i}){Q}_{o}$.

Therefore, in the case of a converging nanojunction, *dN*/*dt* should increase by a factor of about 18.7/11.9 ≈ 3/2, while for the diverging junction *dN*/*dt* must decrease by a factor of 2/3. But, as Fig. 4 indicates, *Q*_*i*_/*Q*_*o*_ ≈ 9000/13000 ≈ 0.7 and ≈2500/5000 = 1/2 for, respectively, the converging and diverging junctions. As mentioned earlier, the fact that in both cases *Q*_*i*_ is much less than the theoretical prediction indicates that the transition region dissipates a significant amount of the fluid’s energy.

An approximate expression for such energy dissipation is derived in the SI based on continuum hydrodynamics. It suggests that the transition region may be viewed as having a hydraulic resistance that causes a pressure drop,

4 $${\rm{\Delta }}{P}_{t}=\frac{\alpha \eta L^{\prime} Q}{\langle {R}^{4}\rangle },$$

where *α* is a numerical constant that depends only on the slope of the junction in the transition region (see the SI), *L*′ is the effective length of the transition region with (*n* − 1) being the number of short CNT rings that make up the junction, and 〈*R*^4^〉 is the mean value of the fourth power of the local radius in the transition region. Note that we do not imply that the carbon rings represent rough surfaces, and that in deriving Eq. (4) we did not consider the variation of the viscosity *η* with the size of the opening. A more precise estimate of Δ*P*_*t*_ would be based on the average shear viscosity of the fluid in the nanojunctions^44,45^. The pressure drop given by Eq. (4) must be added to the one that results from viscous dissipation at the entrance of the CNT, given by Eq. (3). Therefore, when the interface reaches the second straight CNT of radius *R*_*i*_, the Laplace pressure *P*_*c*_ = *β*/*R*_*i*_ that supplies the input power for spontaneous imbibition should be equal to the sum of the two pressure drops. Setting the two equal and solving for *Q* then yields,

5 $$Q=\frac{\beta }{\eta }\frac{1}{{R}_{i}}{(\frac{C}{{R}_{o}^{3}}+\frac{\alpha \eta L^{\prime} }{\langle {R}^{4}\rangle })}^{-1}.$$

There is no transition for a straight CNT and, thus, *L*′ = 0, and *R* = *R*_*o*_ = *R*_*i*_. Therefore, the rate of water uptake *Q*_*s*_ in a straight CNT of radius *R* is, *Q*_*s*_ = *βR*^2^/(*Cη*), which should be compared with flow rate *Q* in a nanojunction, Eq. (5). Therefore,

6 $$\frac{{Q}_{s}}{Q}=\frac{{R}^{2}{R}_{i}}{{R}_{o}^{3}}+\frac{\alpha }{C}\frac{{R}^{2}{R}_{i}}{\langle {R}^{4}\rangle }L^{\prime} \equiv {r}_{1}+{r}_{2}.$$

We point out that in deriving Eq. (6) we ignored the dependence of *C* and *η* on the radius^27,44,45^, and the fact that in narrow tubes the molecular structure of the fluid can affect the dynamics drastically. But, because we only wish to obtain an approximate estimate of the quantities of interest, we have ignored them here.

In Eq. (6) *r*_1_ depends only on the geometry of the opening. Estimating the second term, *r*_2_ = *Q*_*s*_/*Q* − *r*_1_, however, requires all the geometrical details of the transition region. The ratio of the second terms *r*_2_ for a diverging and converging geometry, *r*_2*d*_/*r*_2*c*_ (subscripts *d* and *c* refer to diverging and converging geometries) of the second term for a diverging and converging geometry that consists of the same junctions is independent of *α* and *C* and, hence, it provides us with a simple way of evaluating our assumptions and estimates. Recognizing that we compare the water uptake with the volume flow rate of the *larger* nanotube, for the diverging *d*(20, 30) geometry (i.e., one that connects a (20, 20) and (30, 30) CNTs; see Methods below) we have ${r}_{1d}={R}_{i}^{2}\times ({R}_{i}/{R}_{o}^{3})={R}_{i}^{3}/{R}_{o}^{3}={18.7}^{3}/{11.92}^{3}\approx 3.86$. In the converging *c*(30, 20) geometry, on the other hand, we have, ${r}_{1c}={R}_{o}^{2}\times ({R}_{i}/{R}_{o}^{3})={R}_{i}/{R}_{o}=11.92/18.7\approx 0.64$. Since *α*, Ł′ and 〈*R*^4^〉 are all the same in the CNTs with converging and diverging nanojunctions, we obtain, *r*_2*d*_/*r*_2*c*_ = 18.7/11.92 ≈ 1.57 ± 0.02, where the uncertainty is due to possible variations in the radii. On the other hand, if we use the results of the MD simulation for the straight (30, 30) CNT with *d*(20, 30) and *c*(30, 20) nanojunctions, we obtain, *Q*_*s*_ ≈ 13000 ± 500, *Q*_*d*_ ≈ 2500 ± 500 for the CNT with a diverging nanojunction, and *Q*_*c*_≈9000 ± 900, respectively. Thus, *r*_2*d*_ = *Q*_*s*_/*Q* − *r*_1*d*_ ≈ 1.3 ± 0.3 and *r*_2*c*_ = *Q*_*s*_/*Q* − *r*_1*c*_≈0.8 ± 0.1, which yield *r*_2*d*_/*r*_2*c*_ ≈ 1.6 ± 0.4, in excellent agreement with the theoretical predication. Note that the flow rate is computed by evaluating the slope of the *N*(*t*) curves, where *N*(*t*) is the number of water molecules inside the tube at time *t*, averaged over the number of realizations, which is in most cases is three. The uncertainties are the mean deviations from the average values.

A closer inspection of *N*(*t*) shown in Fig. 4 reveals that Eq. (6) is not valid in the middle flow regime in which water invades the transition region. In the diverging region of the *d*(20, 30) junction, the dynamics of the interface, as well as the uptake, are very slow even when compared with the next stage where the interface moves inside the narrower part. On the other hand, the motion of the interface is a little faster in the converging part of the *c*(30, 20) configuration, such that the flow rate is almost equal to its value during filling of the first segment. These observations suggest that the effect on the motion of the interface in the transition stage depends on its *direction*. To explain this effect, we note that the transition region may be considered as a series of decreasing (increasing) steps of height Δ*R*, with width of the kinks between rings of different diameters being Δ*z* (see Fig. 1). During the passage of water through the transition region the interface encounters a subsequent arrangement of convex and concave edges that are geometrically the same for both converging and diverging configurations. Over microscopic length scales, Gibbs inequality (in terms of the contact angles) implies^46^ that the interface is pinned to the convex edges, whereas it passes through the concave edges. A similar mechanism prevails in the dynamics of interface motion in the transition region in our nanotube system.

**Figure 5:**
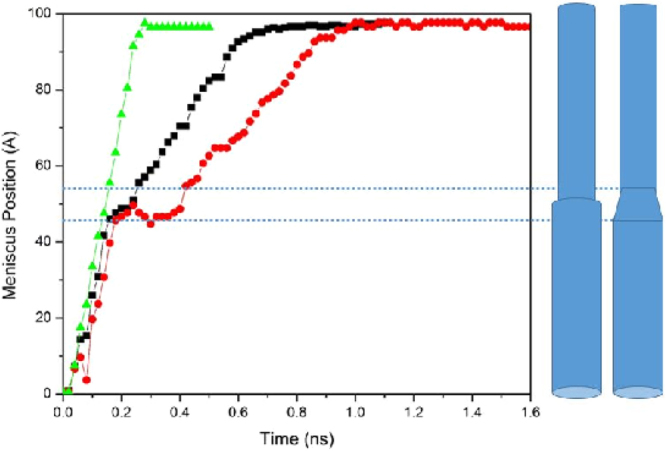
Dynamic evolution of the meniscus position *L*(*t*) for the three-step (black squares) and two-step (red circles) *d*(10, 13) nanojunctions, and its comparison with the corresponding quantity for a straight (13, 13) CNT of the same total length (green triangles).

To further investigate this, we carried out MD simulations with the configurations shown in Fig. 5 that consist of (10, 10) and (13, 13) CNTs of total length of 100 *Å* and radii 5.14 *Å* and 7.17 *Å*, respectively. We refer to the two CNTs with abrupt change of radii as the one-step *d*(10, 13), while the one with three CNTs and diverging junctions is referred to as the three-step *d*(10, 13). Note that, in addition to demonstrating the pining-depinning^47^ of the contact line (see the following discussion), this study also demonstrates the effect of the ‘slope’ of the transition region (how fast or slow it diverges or converges) on the dynamics.

**Figure 6:**
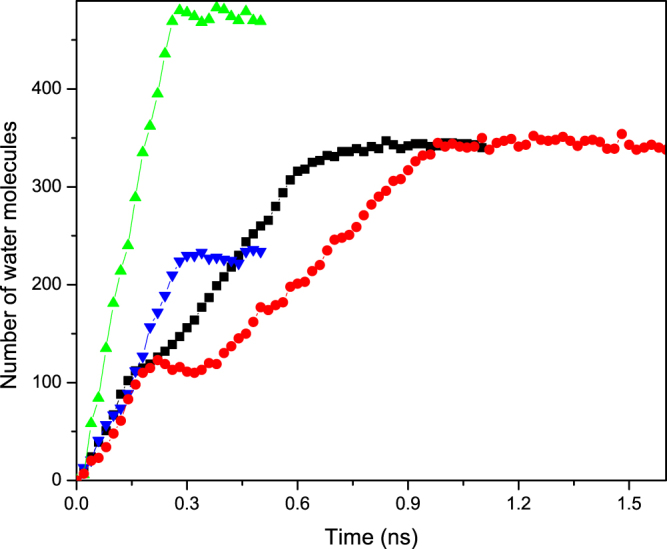
Time-dependence of the number of water molecules *N*(*t*) corresponding to Fig. 4.

Figure 5 compares the evolution of the interface position *L*(*t*) in the two systems with that of a straight (13, 13) CNT. As the figure indicates, the interface is pinned after it reaches the diverging segment. Then, after some time it depins and begins to advance through the junction as a result of the thermal fluctuations^48^. The time interval of the pinning-depinning transition is typically more extended for the two-step junctions. We have previously reported^20^ a similar phenomenon for water uptake in straight, but chemically heterogeneous nanotubes in which the energy interaction parameters between the atoms on the walls of the tube in a small region near the tube’s entrance and the fluid molecules in the tube were smaller than those in the rest of the tube. Figure 6 presents the corresponding water uptakes in the two nanotube configurations, and compares them with those of straight CNTs with the same length.

**Figure 7:**
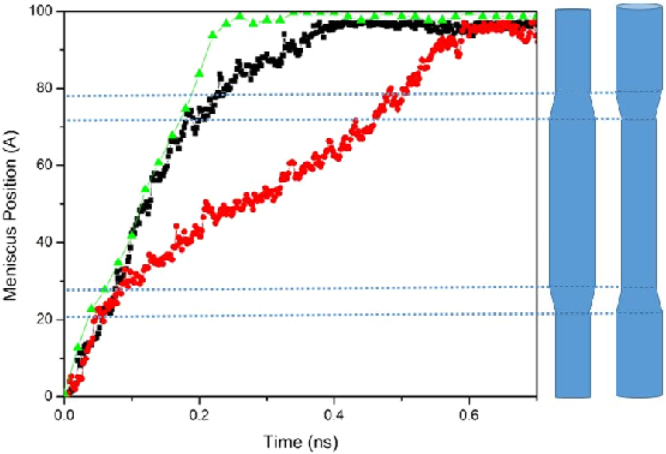
Dynamic evolution of the meniscus position *L*(*t*) for the converging-diverging (black squares) and diverging-converging (red circles) cycles of the 10–13 nanojunctions, and its comparison with the corresponding quantity for a straight (13, 13) CNT of the same total length (green triangles).

Converging and diverging nanojunctions may be combined to produce other functional elements. In microfluidics, a cyclic arrangement that consists of one contraction and one expansion is of great importance due to its applications, because it provides one with a simple way of controlling passively the fluid flow in microchannels^39^. That is, it is possible to enhance mass transfer in narrower channels by connecting it to the reservoir through a CNT of larger opening. This can be particularly desirable, when the size of opening at the downstream must be restricted to certain values. An important aspect of a converging-diverging (CD) or diverging-converging (DC) cycles is that when the size of transition region is negligible and the total length of each microchannel is the same, the total time for filling the cycle is the same for both CD and DC arrangements^34,38^. Our simulations show, however, that this is not the case in the CNTs with nanojunctions. As Fig. 7 indicates, the required time for filling a system with a DC cycle is much larger than that of one with a CD cycle of the same length and volume. As stated earlier, in a nanotube of varying cross section, the size of the channel’s opening determines the entrance friction. As such, in a nanotube with a narrow opening, such as the DC configuration, friction is always greater than in channels with wider opening.

**Figure 8:**
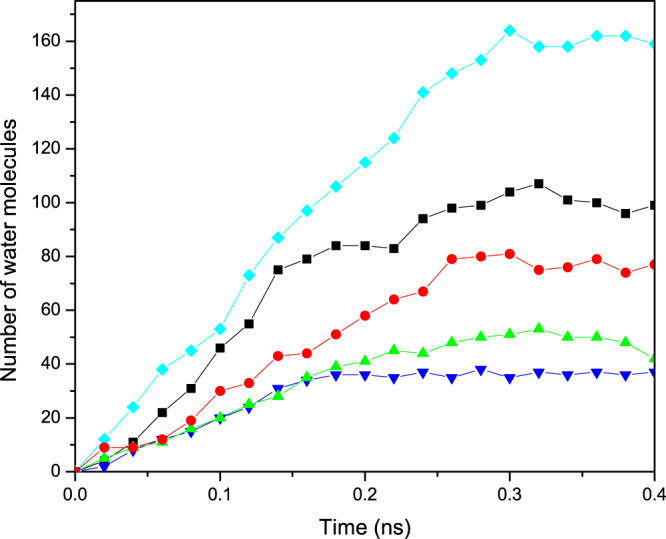
Time-dependence of the number of water molecules *N*(*t*) in the *c*(6, 9) (black squares), *c*(6, 8) (red circles), and *c*(6, 7) (green up triangles) nanojunctions. For comparison, we also show the corresponding quantities for straight (6, 6) (blue down triangles) and (9, 9) (light blue diamond) CNTs of the same total length.

Another remarkable difference between dynamic wetting of nanojunctions and microjunctions is that, the absorption time in the latter depends only on the ratios of the radii and of the lengths. In contrast, dynamic wetting of the nanojunctions is strongly dependent upon the size of *every* straight segment that constitutes the nanojunction. For example, a converging *c*(9, 6) nanojunction has the same radius ratio as a *c*(30, 20) junction. Our MD simulations show, however, that unlike the *c*(30, 20) nanojunctions, the *c*(9, 6) configuration does *not* lead to faster imbibition. This is shown in Fig. 8. Besides, in the transition region the dynamics becomes so slow for a while that it can be regarded as if the interface has been pinned, although we do not expect that to happen at a concave edge. As Fig. 8 indicates, the *c*(8, 6) and *c*(7, 6) nanojunctions do not also result in more rapid interface advancement than a straight (6, 6) CNT. This feature is related to fact that, under both equilibrium and nonequilibrium conditions, the distribution of the fluid molecules inside very small confined media is highly structured whose details depend on the tube’s radius^9,48^.

Finally, let us point out that it is possible to investigate how the change in the wettability of CNTs affects the outcome of flow in the type of systems that we study. Theoretically, the wetting properties of CNT can be tuned by changing the water-carbon interaction parameters. For example, in the case of a diverging junction consisting of a (10, 10) and a (13, 13) CNT, the rate of mass uptake in the first and final stages is *Q*_*i*_ = 670 ± 30 and *Q*_*o*_ = 520 ± 10, respectively. We increased the strength of Lennard-Jones interaction between water and carbon atoms by only 10% and carried out a series of simulations. The result was that the rate of mass uptake increased drastically: *Q*_*i*_ = 1600 ± 300 (240 ± 50% increase), and *Q*_*o*_ = 1100 ± 100 (210 ± 20% increase). Equation (5) indicates that the Laplace pressure increases by a factor larger than two, when we increase the strength of the water-carbon interaction by only 10%. Moreover, in a separate simulation, we found that with the given modified water-carbon interaction, a water droplet completely wets a single graphene sheet at *T* = 300 K. It is of course possible that the change in the interaction parameters affects the hydrodynamic resistance in the transition region. As our results indicate, however, the change, if any, is small when compared with the change in the Laplace pressure.

## Summary

Molecular dynamics simulation of water imbibition in nanostructures that consist of CNTs of various sizes, connected together by converging or diverging nanojunctions indicates that, whereas the dynamics of water uptake in the entrance CNT is governed by the model for imbibition in CNTs in which the main source of energy dissipation is the friction at the entrance, the governing equation describing water uptake in the exit CNT is more complex, due to significant energy loss in the nanojunction. A significant difference between dynamic wetting of nano- and microjunctions is that, whereas absorption time in the latter depends only on the ratios of the radii and of the lengths of the channels, the same is not true about the former. Our MD simulation indicate thats water uptake in nanojunctions is strongly dependent upon the size of every segment that constitutes the nanojunction. Simulation of imbibition in nanostructures with cyclic configurations of nanojunctions indicates that the dynamics is much slower when three CNTs are connected by, first, a diverging and then a converging nanojunction than the opposite case of a converging nanojunction followed by a diverging one of the same volume and nanotubes. Interface pinning-depinning also occurs in the convex edges. These results indicate the sensitivity of water imbibition and flow to the structure of nanostructured materials, which can be fruitfully taken advantage of for various applications in nanofluidics and nanoreactors.

## Methods

We first describe the nanotube systems that we study, and then explain the MD simulation procedure.

### Nanotubes with junctions

We investigate mostly two configurations of coaxial CNTs with nanojunctions of total length 100 *Å*. Each configuration consists of two armchair CNTs with chiralities (*n*, *n*) and (*m*, *m*) and a gradual change of the effective radius from *R*_1_ = 3*nd*/(2*π*) − *σ*_C−O_/2 to *R*_2_ = 3*md*/(2*π*) − *σ*_C−O_/2, where *d* = 1.42 *Å* is the length of C-C bond, and *σ*_C−O_ is the Lennard-Jones (LJ) size parameter for the interaction between carbon and oxygen atoms of water. The transition region between the two straight CNTs consists of several concentric CNT rings with axial length of 2.5 *Å*. If the chirality of each CNT differs from the chirality of the next by one unit, as shown in Fig. 1, the resulting geometry is a *k*-steps junction with *k* = |*n* − *m*|. We call such configuration *c*(*n*, *m*) and *d*(*n*, *m*), depending on where the nanojunction converges (*c*) or diverges (*d*) or, equivalently, on the sign of the slope of the variation of the radius in the contracting or the expanding region of the nanojunctions. As Fig. 1 indicates, the slope of variation of *R* with *z* is negative for a converging *c*(*n*, *m*) configuration and positive for a diverging *d*(*n*, *m*). We also examined some other nanojunctions with an abrupt change of radius in the transition region between two main nanotube, as described earlier. The length of the straight CNT through which water entered the system was 25 *Å*, while that of the exit nanotube segment was 50 *Å*.

### Molecular dynamics simulation

The simulations were performed in the (*NVT*) ensemble using the LAMMPS package. The timestep was 2 fs, and all the simulations were at 300 K. We used the TIP3P model of water^49^. We used the Amber-96 force field, which has been used extensively in the past to represent water-carbon interaction in CNTs^26,50,51^.

Although care was taken to ensure that the thermostat did not impart artificial motion to the flow, but because we are dealing with a dynamic phenomenon, its possible effect on the results must be carefully examined. Thus, to check that the thermostat did not give rise to any unphysical effect, we also carried out additional computations with a (20, 20) CNT in which the simulations began in the (*NVT*) ensemble and the temperature was set at 300 K. After equilibrium was reached, the thermostat was removed and the calculations continued in the (*NVE*) ensemble. As documented in the SI, the temperature throughout the nanotube did not change significantly in the (*NVE*) ensemble; we calculated it to be (using the standard technique based on the kinetic energy), *T* ≈ 305 ± 13 K, which is very close to set temperature of 300 K in the (*NVT*) ensemble. In addition, the flux *dN*/*dt* (where *N* is the number of the water molecules) in the same CNT was estimated to be, *dN*/*dt* ≈ 5100 ± 900 after equilibrium had been reached in the (*NVT*) ensemble, while it was *dN*/*dt* ≈ 5700 ± 700 in the (*NVE*) ensemble that followed the calculations in the (*NVT*) ensemble. Thus, our results presented in this paper, which were obtained by carrying out MD simulations in the (*NVT*) ensemble, were not affected significantly by the presence of the thermostat.

Since we study water imbibition in small CNTs, the contact angle (CA) of water with the internal surface of the nanotubes is important. The CA of water on the surface of CNTs has been reported to be^26,50,51^, *θ* ≈ 57°, which is in the middle of the range of the experimental values of the CA on graphite^52^. Using MD simulation, we also computed the CA for water droplets in (20, 20) and (30, 30) CNTs, using the method proposed by Werder *et al*.^53^. The details are given in the SI.

At the beginning of the simulation, the water molecules were disposed on a simple cubic lattice in a reservoir that consisted of two graphene sheets perpendicular to the nanotubes’ axis, with the left end of the entrance CNT capped, while the right end remained open during the entire simulation; see Fig. 1. The density of water at 300 K was 0.98 gr/cm^3^. When the water distribution in the reservoir reached equilibrium, after typically 100 ps, the cap was removed to allow it to imbibe into the entrance CNT. For the carbon-water interaction we used the standard 6–12 LJ potential for the oxygen-carbon interaction with^1,6^ ${\epsilon }_{{\rm{C}}-{\rm{O}}}=114.4$ cal/mol for the energy parameter and *σ*_C−O_ = 3.276 *Å*, with a cutoff of 14 *Å*. We used the particle-particle particle-mesh (PPPM) method^54,55^ for computing long-range Coulombic interactions with a cutoff of 10 *Å*. More details of the simulation procedure were given elsewhere^18^.

## Electronic supplementary material

Water Flow in Carbon Nanotubes with Nanojunctions: Dissipation, Interface Pinning and Fast Dynamics
