# Supplementary material for: Nanojunction Effects on Water Flow in Carbon Nanotubes
Source: Sci Rep. 2018 May 17;8:7752. doi: 10.1038/s41598-018-26072-6 (PMC5958144; doi:10.1038/s41598-018-26072-6)
Supplement: Supplementary file 1 — Water Flow in Carbon Nanotubes with Nanojunctions: Dissipation, Interface Pinning and Fast Dynamics [file 41598_2018_26072_MOESM1_ESM.pdf]

Supplementary information for

Nanojunction effects on water flow in carbon nanotubes

Fatemeh Ebrahimi, Farzaneh Ramazani, & Muhammad Sahimi

Viscous dissipation. We derive an expression for viscous dissipation in a nanojunction of the type shown in Figure 8 of the main text of the paper. Viscous dissipation per unit volume φ_v is given by, $\boldsymbol{\tau}:\nabla\boldsymbol{v} = \eta\varphi_v$, where $\boldsymbol{\tau}$ is the stress tensor, $\boldsymbol{v} = (v_r, v_\theta, v_z)$ is the velocity vector of the fluid, and η is the fluid's viscosity. For a Newtonian fluid flowing in a system with cylindrical coordinates, φ_v is given by,

$$\varphi_v = 2\left[\left(\frac{\partial v_r}{\partial r}\right)^2 + \left(\frac{1}{r}\frac{\partial v_\theta}{\partial \theta} + \frac{v_r}{r}\right)^2 + \left(\frac{\partial v_z}{\partial z}\right)^2\right] + \left[r\frac{\partial}{\partial r}\left(\frac{v_\theta}{r}\right) + \frac{1}{r}\frac{\partial v_r}{\partial \theta}\right]^2 + \left[\frac{1}{r}\frac{\partial v_z}{\partial \theta} + \frac{\partial v_\theta}{\partial z}\right]^2 + \left[\frac{\partial v_r}{\partial \theta} + \frac{\partial v_z}{\partial r}\right]^2 - \frac{2}{3}(\vec{\nabla}.\vec{v})^2 \qquad \textbf{(1)}$$

The last term of the right side of Eq. (1) vanishes due to incompressibility of water. To evaluate the dissipation function by Eq. (1), we use the time-averaged fluid velocities. v_r does not vanish due to the slope of the variation of the radius R with the axial position, $dR\,/\,dz$. Given that $v_\theta = 0$ and neither v_z nor v_r depends on the angular variable θ, the expression for φ_v is simplified to,

$$\varphi_v = 2\left[\left(\frac{\partial v_r}{\partial r}\right)^2 + \left(\frac{v_r}{r}\right)^2 + \left(\frac{\partial v_z}{\partial z}\right)^2\right] + \left(\frac{\partial v_z}{\partial r}\right)^2 \qquad \textbf{(2)}$$

Consider Figure 8 of the main text of the paper. As described in the paper, a nanojunction consists of n short carbon nanotubes (CNTs) of decreasing (or increasing) radii $R(z)$ whose axes are aligned with the z direction. Hereafter, we refer to such short CNTs as rings. Thus, if Q is the volume flow rate, then,

$$v_z(z) = \frac{Q}{\pi R(z)^2} \quad \textbf{(3)}$$

Using the well-known lubrication approximation, we obtain an approximate expression for v_r:

$$v_r \approx v_{max} \frac{r}{R(z)} \approx \frac{\Delta R}{\Delta t} \frac{r}{R(z)} \quad \textbf{(4)}$$

where Δt is the time needed for a molecule with axial velocity $v(z)$ to pass through the nth ring of a nanojunction. Assuming that both ΔR and Δt are small, we obtain, $\Delta t = \frac{\Delta z}{v_z}$ and, therefore,

$$v_r \approx \frac{\Delta R}{\Delta z} \frac{v_z}{R(z)} r \quad \textbf{(5)}$$

Substituting for v_r and v_z in Eq. (2) and summing over all the n rings of a nanojunction yields,

$$\varphi_v = \alpha Q^2 \frac{L'}{< R^4 >} \quad \textbf{(6)}$$

in which

$$\alpha = \frac{\eta}{\pi} \left(\frac{\Delta R}{\Delta z}\right)^2 \left[12 + \frac{9}{2}\left(\frac{\Delta R}{\Delta z}\right)^2\right] \quad \textbf{(7)}$$

depends only on the slope $\frac{\Delta R}{\Delta z}$, and $L^{'} = n\Delta z$ is the effective length of the transition zone. Here,

$$\frac{1}{< R^4 >} = \sum_n \frac{1}{R_n^4} \quad \textbf{(6)}$$

with R_n being the radius of the nth ring. This is equivalent to a pressure dropΔP_t, given by

$$\Delta P_t = \frac{\alpha \eta L' Q}{\bar{R}^4} \quad \textbf{(9)}$$

resulting from the change in the radius in the transition region, which is Eq. (4) of the main text of the paper.

Assessing the effect of the thermostat on the results. As described in the main text of the paper, our molecular dynamics (MD) simulations were performed in the(NVT) ensemble. When dealing with a dynamic phenomenon, however, a thermostat might give rise to spurious effects. Thus, to ensure that the thermostat did not give generate any unphysical effect, we also carried out MD computations witha (20,20) CNT in which the simulations began in the (NVT) ensemble. After equilibrium was reached, the thermostat was removed and the simulations continued in the (NVE) ensemble. We computed two important properties in order to assess the effect of the thermostat.

(i) During the simulations in the (NVT) ensemble, the thermostat set the temperature T at 300 K. Therefore, we computed the (average) temperature throughout the nanotube during the simulations in the (NVE) ensemble, in order to see whether Tdeviates significantly from its set value of 300 K. Figure S1 presents the variations of temperature inside the (20,20) CNT as a function of the meniscus position in the (NVE) ensemble, which were computed after equilibrium had been reached in the (NVT) ensemble and the thermostat had been removed. The average temperature with the (NVE) ensemble is, $T \approx 305 \pm 13$ K, very close to 300 K set by the (NVT) ensemble.

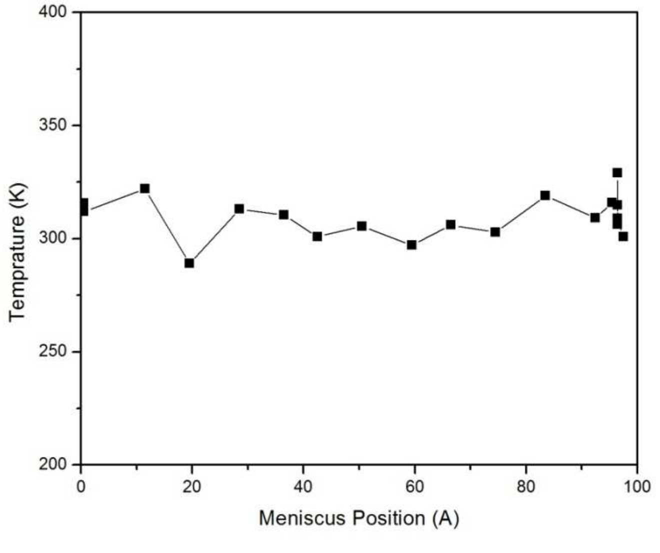

Figure S1: Variations of temperature inside a (20,20) CNT as a function of the meniscus position in the (NVE) ensemble. The average temperature is throughout the tube is, 305 ± 13 K.

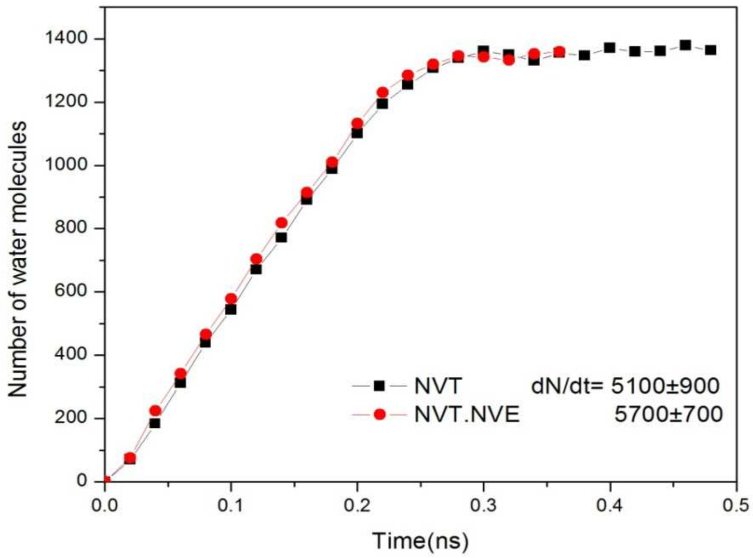

Figure S2: Dependence on time of the number of water molecules N in a (20,20) CNT, computed by two ensembles, both averaged over three realizations. Black squares indicate the results obtained by the (NVT) ensemble, while red circles denote the results obtained in the (NVE) ensemble after equilibrium had been reached in the (NVT) ensemble.

(ii) We also computed the flux dN/dt, where N is the number of the water molecules. Figure S2 presents the results. After the simulation reached equilibrium in the ensemble, we estimated that, $dN/dt \approx 5100 \pm 900$, while $dN/dt \approx 5700 \pm 700$, in the (NVE) ensemble that followed the calculations in the (NVT) ensemble. The two estimates are completely consistent. Thus, the results presented in the paper, which were computed by carrying out the MD simulations in the (NVT) ensemble, were not affected significantly by the presence of the thermostat.

Calculation of the contact angle of water with the nanotube's wall. We also carried out MD simulations to estimate the contact angle (CA) of nanometer-size water droplets with the surface of the CNT, and its possible dependence on the tubes' geometry. To do so, we used the method proposed by Werder[1], which is based on the least square fits of the isochore lines, lines of constant density, to a circle (or sphere). In this method the equilibrium configurations of a water nanodroplet at the desired temperature are used to compute the isochore lines at various levels.

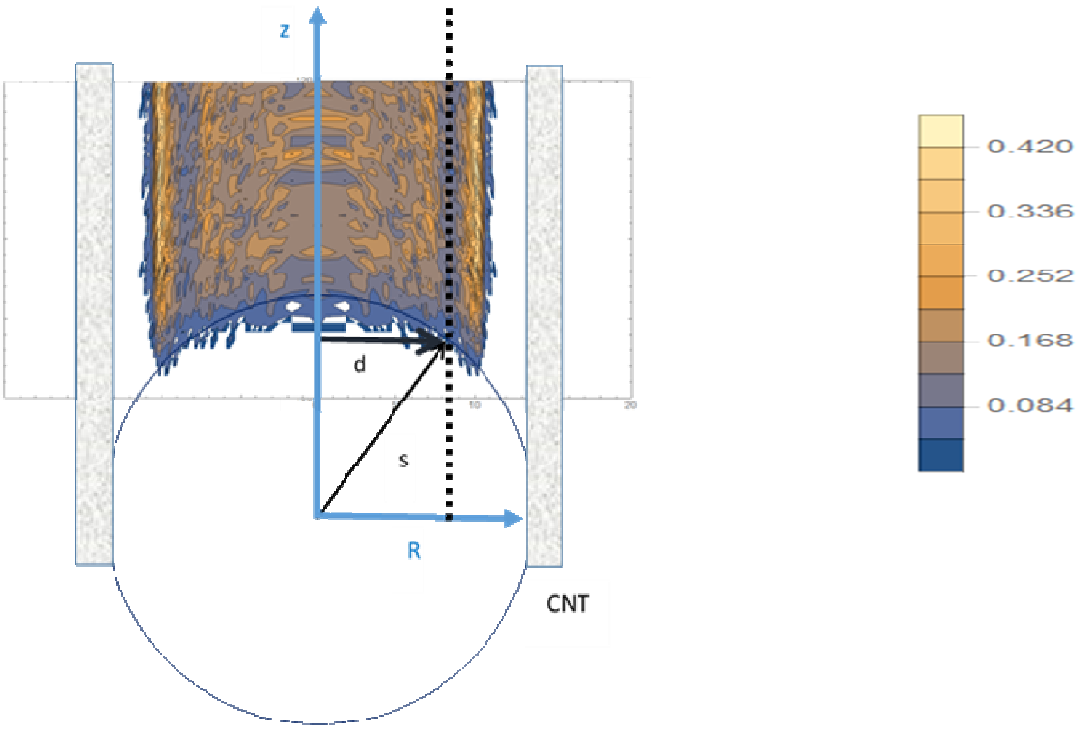

Figure S3: The isochors for a water droplet in a (20,20)CNT, averaged over 3.5 ns (the color bar is for water density in gr/cm^3. The contact angle is determined from the radii d and s through the relation, $\theta = \pi - cos^{-1}(\frac{d}{s})$, where d is the (half) chord that excludes the thin water layer near the wall, and s is the radius of the fitted circle (sphere).

After reaching equilibrium and ignoring a thin layer of adsorbed water molecules, as described by Bormashenko[2], the least-square fit of the isochore lines is superimposed on the figure, from which the CA is determined by a simple geometrical construction. This is shown in Figure S3. Our calculations indicated that the CA in the (20,20) CNT is (in degrees) 55 ± 8; see the isochores shown in Figure S3. The CA in the (30,30) CNT turned out to be 54 ± 7. The relatively large fluctuations are due to the small radii of the CNTs. We also computed the CA in a converging configuration, shown in Fig. S4, with the corresponding isochore lines shown in Figure S5. The CA turned out to be 59 ± 8. Thus, the CAs for the various configurations are consistent with each other, and are also in agreement with what has been reported in the literature.

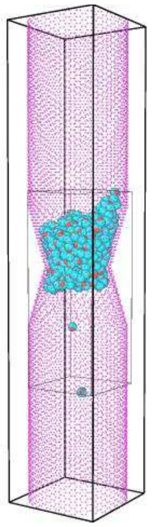

Figure S4: A snapshot of water droplet in the configuration that converges at the center.

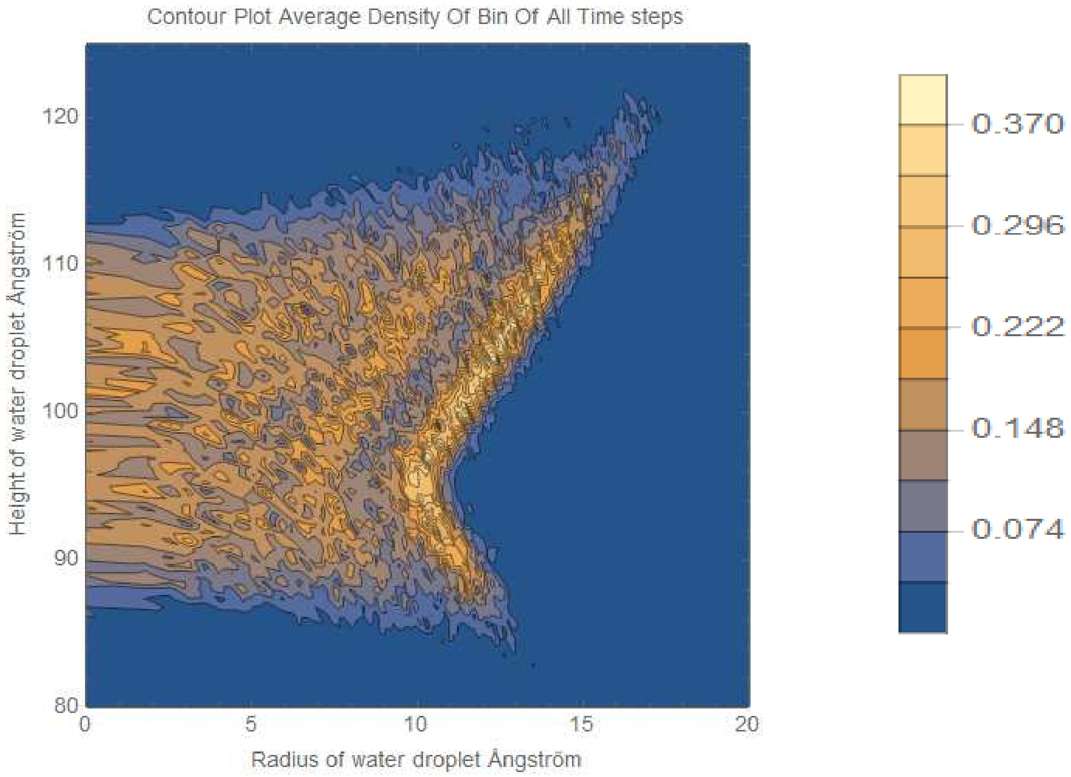

Figure S5: The isochors for water droplet, averaged over 3.5 ns, which correspond to Figure S4.

References

[1] Werder, T., Walther, J.H., Jaffe, R.L., Halicioglu, T., Noca, F., & Koumoutsakos, P. Molecular dynamics simulation of contact angles of water droplets in carbon nanotubes. *Nano Lett.* **1**, 697-702 (2001).

[2] Bormashenko, E.Y. *Wetting of Real Surfaces*, (Walter de Gruyter GmbH, Berlin, 2013).
